# Injectable Brain Extracellular Matrix Hydrogels Enhance Neuronal Migration and Functional Recovery After Intracerebral Hemorrhage

**DOI:** 10.34133/bmr.0192

**Published:** 2025-04-22

**Authors:** Jiajie Xia, Xinjie Gao, Jun Yao, Yuchao Fei, Dagang Song, Zhiwei Gu, Gang Zheng, Yuxiang Gu, Chuanjian Tu

**Affiliations:** ^1^ Department of Neurosurgery, Neurosurgery Research Institute, The First Affiliated Hospital, Fujian Medical University, Fuzhou, Fujian 350005, China.; ^2^ Department of Neurosurgery, Shaoxing Central Hospital, The Central Affiliated Hospital, Shaoxing University, Shaoxing, Zhejiang 312030, China.; ^3^ Department of Neurosurgery of Huashan Hospital, State Key Laboratory of Medical Neurobiology, MOE Frontiers Center for Brain Science, and Institutes of Brain Science, Fudan University, Shanghai 200000, China.; ^4^ Department of Orthopedic Surgery, Shaoxing Central Hospital, The Central Affiliated Hospital, Shaoxing University, Shaoxing, Zhejiang 312030, China.; ^5^ Department of Neurosurgery, Shaoxing Central Hospital, China Medical University, Shaoxing, Zhejiang 312030, China.

## Abstract

Neural repair within the lesion cavity caused by intracerebral hemorrhage (ICH) remains a major therapeutic challenge. Hydrogels hold great potential in regenerative medicine as functional scaffolds. However, inadequate host cell infiltration and suboptimal delivery methods have limited their application in tissue engineering. Here, we describe an optimized decellularization approach to create injectable brain extracellular matrix (ECM) hydrogels for the treatment of ICH. The hydrogel exhibits excellent biodegradability and biocompatibility. In an ICH rat model, the hydrogel implanted into the stroke cavity promoted neural recovery, facilitated cell recruitment, enhanced angiogenesis, and inhibited inflammation in the peri-cavity region at 14 d post-implantation. Furthermore, the hydrogel improved cell proliferation and migration, reversed cell apoptosis, and modulated transcriptomic changes in vitro. Notably, the hydrogel may promote neuronal migration and neural functional recovery after ICH through the slit guidance ligand 2–receptor roundabout guidance receptor 1 (Slit2-Robo1) signaling pathway. These findings highlight the potential of brain ECM hydrogels as a promising strategy for brain tissue regeneration.

## Introduction

Intracerebral hemorrhage (ICH) constitutes the second predominant stroke, representing 10% to 40% of all incident cases [1–4]. Patients with ICH often suffer from neurological damage and disability, which hinders their independence and results in substantial social burdens and insurance costs. The rapid formation of hematoma in the brain parenchyma disrupts the brain tissue. The nerve bundles surrounding the hematoma are displaced and deformed due to the occupancy. Although hematomas typically resolve over time, stroke creates cavities accompanied by neurological dysfunction [5]. Despite a considerable survival rate after the initial onset, the continued hemorrhage-induced secondary effects can lead to severe neurological impairment and fatality [6]. After ICH, astrocytes adjacent to the hematoma transform into reactive astrocytes, rapidly responding to injury signals and forming a dense glial scar around the lesion site in collaboration with oligodendrocyte progenitor cells (OPCs) and microglia. As a physical barrier, the glial scar inhibits the regeneration of nerve fibers, preventing them from crossing the lesion and reconnecting with their original targets. Additionally, certain extracellular matrix (ECM) molecules secreted within the glial scar tissue have been shown to actively suppress axonal growth. Although promoting brain tissue repair post-ICH is considered a potential therapeutic strategy, the early formation of the glial scar introduces significant challenges to neural repair.

ECM isolated from tissues exhibits considerable potential for biomedical purposes, notably in regenerative medicine. The distinctive composition of ECMs from different organs suggests that ECM constructs derived from specific tissue types may contain unique bioactive molecules capable of stimulating constructive transformation in those tissue types [7]. In nonlesioned tissue, the brain ECM, primarily consisting of collagen, hyaluronic acid, proteoglycans, and glycoproteins, demonstrates significant potential to facilitate early neural repair [8,9]. These constituents contribute to various physiological phenomena involved in neurogenesis, including neuron polarization and migration, synaptogenesis, axon guidance, and synapse maturation, and have been used for site-specific differentiation effects of neuronal stem cells and induced pluripotent stem cells [10,11]. Studies related to Parkinson's disease, brain organoids, neuroinflammatory responses, glioma, and traumatic brain injury have also suggested [9,12–15] that brain ECM exerts heightened efficacy on brain-sourced cells [16] and stimulates central nervous system tissue rebuilding [17], exhibiting tissue-specific outcomes. The brain ECM may contribute to neuroprotection in central nervous system diseases and promote brain regeneration. However, even when injected directly into the brain, ECM powders or sheets degrade rapidly, making it challenging to achieve sustained, continuous repair-promoting effects.

Currently, preclinical studies have shown that injection of biological scaffolds into the hematoma cavity can be used as a potential pro-repair approach [18,19]. Hydrogels, composed of materials such as gelatin [20], keratin [21,22], silk fibroin [23], peptides [24], and hyaluronan and chitosan [25], are commonly used as drug delivery carriers and biological scaffolds. Their slow-degrading nature allows hydrogels to remain in body cavities for extended periods. The hydrogel provided structural support to fill the cavity formed after ICH and created an adaptable environment that allowed for cell spreading and migration [24–26].

Although hydrogels tested in ICH models have demonstrated certain regenerative potential, several limitations have hindered their widespread application. Traditional hydrogels often lack bioactivity and fail to provide the necessary biochemical cues to promote host cell infiltration and tissue integration [20]. Natural hydrogels may degrade too rapidly, limiting their ability to support long-term regeneration [21], while non-injectable hydrogels are inconvenient for minimally invasive surgery and struggle to adapt to irregular defects [27]. Additionally, some hydrogels exhibit insufficient host cell infiltration due to the nonspecificity of their components and the absence of brain-specific ECM cues, which are critical for guiding cellular behavior. To address these limitations, we utilized a brain-derived ECM hydrogel, which inherently contains brain-specific biochemical and biophysical properties. This hydrogel provides a 3-dimensional (3D) structure that mimics the brain tissue microenvironment, supporting the spatial distribution and connection of neural cells.

**Fig. 1.**
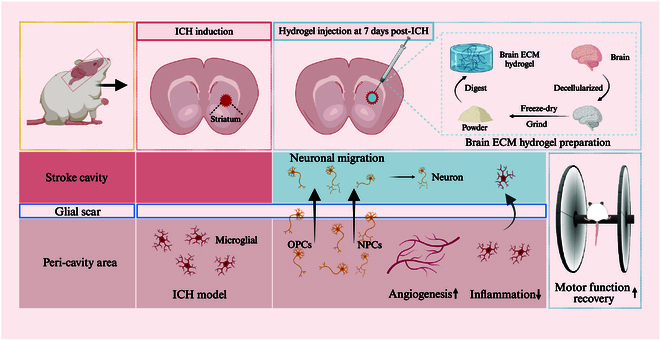
Schematic representation of the preparation and application of brain ECM hydrogels. ECMs were derived from fully decellularized brain tissue. The brain ECM hydrogels were subsequently synthesized and administered via in situ injection to facilitate motor function recovery, promote cellular infiltration into the hydrogel, stimulate angiogenesis in the peri-cavity region, and attenuate inflammation in the peri-cavity region.

We hypothesize that injecting brain ECM hydrogels into the lesion cavity during the recovery period after ICH may accelerate neural repair through multiple mechanisms. The hydrogel provides a biomimetic microenvironment that supports cell adhesion, proliferation, and differentiation, thereby promoting endogenous neural stem cell recruitment and tissue regeneration. Its slow degradation profile ensures that it remains in place for several weeks, providing structural support while gradually being replaced by native tissue. Furthermore, the hydrogel enhances angiogenesis, inhibits the inflammatory response in the peri-cavity area, and recruits endogenous cell migration, all of which are critical for long-term brain tissue recovery. By modulating these key processes, the hydrogel not only accelerates neural repair but also supports functional restoration after ICH. Additionally, we aim to preliminarily explore the signaling pathways modulated by these hydrogels, potentially providing insights for further enhancing their efficacy (Fig. 1).

## Materials and Methods

### Preparation of brain ECM hydrogels

Porcine brain tissues were obtained from 6-month-old Duroc × Landrace × Yorkshire (DLY) pigs, a common commercial hybrid breed. The cerebrum was carefully dissected and frozen at −80 °C immediately after harvest and then thawed before use. The brain was subjected to successive decellularization baths: deionized water (120 min; 300 rpm), 1.0% Triton X-100 (60 min; 120 rpm), deionized water (10 min; 120 rpm), 4.0% deoxycholate (60 min; 120 rpm), 0.1% peracetic acid in 4.0% ethanol (v/v; 120 min; 120 rpm), deionized water (10 min; 120 rpm), and phosphate-buffered saline (PBS) (10 min; 120 rpm). Following each bath, the tissue remnants were thoroughly rinsed with deionized water using a filter. To assess residual DNA, we fixed the brain and nonlyophilized ECM in 10% neutral buffered formalin, which was then embedded in paraffin, sectioned, and stained with hematoxylin and eosin (H&E) or with 4′,6-diamidino-2-phenylindole (DAPI). DNA Extraction Kit (TaKaRa) was used in accordance with the manufacturer’s instructions to quantify the double-stranded DNA.

The ECM samples were subjected to freeze-drying at −80 °C under a vacuum of 0.2 mbar for 48 h using a freeze-dryer (LABCONCO, Kansas City, MO, USA) and subsequently stored at −80 °C until further use. Subsequently, the ECM was finely ground into powder smaller than 300 mesh size. The ground ECM was added to a 0.01 N HCl solution containing 1 mg/ml pepsin (Sigma) at a concentration of 10 mg/ml ECM and stirred at room temperature for 48 h, forming a pre-gel ECM solution with fluidity. NaOH solution (0.1 M) was used to modify the pH of the pre-gel ECM solution to 8.1, rendering pepsin inactive and regulating the hydrogel osmotic pressure using PBS.

### Animals

Sprague–Dawley rats (male, 280 to 320 g) were purchased from the Zhejiang Center of Laboratory Animals (Hangzhou, China). All experimental protocols were reviewed and approved by the Institutional Animal Care and Use Committee (approval no. ZJCLA-IACUC-20010059). Rats were given ad libitum access to food and water. All operations were conducted by experienced staff, and every endeavor was implemented to reduce pain.

### ICH model in vivo

Sprague–Dawley rats were anesthetized using isoflurane inhalation and fixed on a stereotactic frame. A 26-gauge needle was inserted through a burr hole into the striatum (0.5 mm anterior to bregma, 3.5 mm laterally, and 4.5 mm ventral to the dura mater). Fifty microliters of autologous whole blood was infused at 2 μl/min by a microinfusion pump. The needle was held for 5 min after the injection and slowly withdrawn to prevent backflow. Rats in the sham-operated group underwent the same anesthesia and exposure of the brain without injection. As preliminary experiments showed that by 7 d after ICH, the hematoma was partially absorbed, forming a cavity suitable for hydrogel injection, rats suffering from ICH underwent the second operation at this time. Freshly prepared hydrogel was then injected into the striatum cavity of these ICH rats at a dosage of 20 μl. The ICH rats treated with 20 μl of PBS were used as ICH + PBS groups.

### Animal behavioral test

Pretraining was executed 3 d before surgery, and performance was recorded as a baseline. Assessments were conducted on postoperative days 7, 14, and 21. The behavior tests were assessed by the same assessor who was blinded to the group randomization. Repeated-measures analysis of variance (ANOVA) was used to compare the means across different time points (postoperative days 7, 14, and 21).

#### Corner test

The experimental arrangement comprises 2 plates at a 30° angle, with a narrow gap between the 2 plates to encourage the animal to move toward the corner. The rat was allowed to turn around in a direction of its own choice. Each rat was performed with 10 repetitions, with an interval of 1 min. Subsequently, turns to the left were tallied.

#### Rotarod test

The rats were placed on the rods of the accelerating rotarod, and the time the animals remained on the rod was measured. The rotarod was set to accelerate from zero to 40 rpm in 120 s. To ensure data reliability, each rat was tested 3 times with a 10-min interval between trials. Soft cushions were positioned beneath the rod to prevent harm to the animal.

#### Foot-fault test

The rats were allowed to walk along a horizontal ladder with rungs (2 cm apart). A foot fault was considered when one of the limbs was suspended inside one of the holes in the metal grid. For each rat, foot faults were recorded. The foot-fault rate was calculated as the number of wrong steps divided by the number of total steps. Each rat was tested 3 times with a 20-min interval between trials to minimize fatigue effects.

### Immunohistochemistry analysis

To analyze the distribution of the ECM hydrogel and the cell infiltration within the hydrogel, 6 rats per group were anesthetized with sodium pentobarbital (50 mg/kg, intraperitoneally) and transcranially perfused with 0.1 M PBS, followed by 4% paraformaldehyde (PFA) in 0.1 M PBS. The transcranial perfusion was performed by first inserting the needle into the left ventricle of the heart and gradually infusing the perfusion solutions. Brains were carefully dissected and postfixed in 4% PFA in PBS for at least 1 day at 4 °C. The brains were then dehydrated in a sucrose gradient series (10%, 20%, 30%) and frozen using Optimal Cutting Temperature (OCT) compound (Sakura) for cryosectioning. Coronal brain sections (10 μm thick) were sliced using a cryostat.

The sections were incubated with Immunol Staining Blocking Buffer (Beyotime) for 1 h at 24 °C, followed by incubation for 16 h at 4 °C with primary antibodies diluted in Immunol Staining Primary Antibody Dilution Buffer (Beyotime). The sections were then incubated for 1 h at 24 °C with secondary antibodies [Alexa Fluor 488, 555, and 647 (1:1,000, Beyotime)]. The details of the primary antibodies are listed in Table. Antibody visualization was performed using a Nikon Ts100-F fluorescence microscope and a Nikon A1 confocal microscope.

**Table.** List of primary antibodies

Antibody (host)	Dilution ratio	Company	Cat. ref.
GFAP (rabbit)	1:1,000	Proteintech	16825-1-AP
GFAP (mouse)	1:500	Proteintech	60190-1-Ig
Laminin β1 (rabbit)	1:500	Proteintech	23498-1-AP
VWF (rabbit)	1:500	Proteintech	27186-1-AP
VEGFA (mouse)	1:500	Proteintech	66828-1-Ig
Nestin (rabbit)	1:500	Proteintech	19483-1-AP
SOX2 (mouse)	1:500	Proteintech	66411-1-Ig
NeuN (rabbit)	1:500	Proteintech	26975-1-AP
ROBO1(rabbit)	1:500	Proteintech	20219-1-AP
DCX (mouse)	1:200	Santa Cruz	sc-271390
IBA1 (goat)	1:1,000	Invitrogen	PA5-18039
NG2 (rabbit)	1:500	Proteintech	55027-1-AP

The percentage of positive cells was calculated through semiquantification of immunofluorescence images. Positive cells were manually counted within defined regions of interest (ROIs) in both the stroke cavity and peri-cavity areas, with the boundary of the stroke cavity identified by the accumulation of glial fibrillary acidic protein–positive (GFAP^+^) astrocytes. The peri-cavity area was defined as the tissue within a radial distance of 200 to 300 μm from the edge of the hematoma cavity. The total number of cells in the ROI was determined using DAPI staining. The percentage of positive cells was calculated by dividing the number of positive cells by the total number of cells in the ROI and then multiplying by 100. Image analyses were performed on 1 or 2 randomly selected microscopic fields in the stroke cavity areas of the striatum in each section, with 2 sections per rat brain. The images were processed and quantified using ImageJ software (National Institutes of Health) by 2 independent observers who were blinded to the experimental groups. Results were expressed as the mean percentage of positive cells, based on data from at least 2 sections per rat brain.

### Quantitative reverse transcription polymerase chain reaction

Four rats per group were used for quantitative reverse transcription polymerase chain reaction (RT-qPCR) analysis to assess gene expression levels. Total RNA was extracted from the ipsilateral striatum of rats on the 21st day post-ICH or from sham-operated rats using TRIzol Reagent (Thermo Fisher Scientific) according to the manufacturer’s protocol. RNA purity and concentration were determined using a NanoDrop ND-2000 spectrophotometer (Thermo Fisher Scientific), and RNA integrity was confirmed by agarose gel electrophoresis. Only RNA samples with an *A*_260_/*A*_280_ ratio between 1.8 and 2.0 were included in subsequent analyses. RNA was reversely transcribed into cDNA using the RevertAid First Strand cDNA Synthesis Kit (Invitrogen). Primers for Slit2 and Robo1 were designed using National Center for Biotechnology Information (NCBI) Primer-BLAST, ensuring the absence of secondary structures and off-target effects (Table S1). RT-qPCR was performed using a QuantStudio 5 Real-Time PCR System (Thermo Fisher Scientific) and qPCR SYBR Green Master Mix (Yeasen). The relative expression levels of Slit2 and Robo1 were normalized to Gapdh expression and calculated using the 2^−ΔΔCt^ method. Results were expressed as fold changes relative to the control group. All RT-qPCRs were performed in triplicate, with at least 4 biological replicates per group. Tissue samples were obtained following euthanasia performed under deep isoflurane anesthesia, followed by transcardial perfusion with 0.1 mM PBS. Ipsilateral striatum tissues were carefully dissected, flash-frozen in liquid nitrogen, and stored at −80 °C for subsequent RNA extraction. All procedures involving animals were approved and conducted in accordance with institutional animal care and use guidelines.

### Protein preparation and Western blotting

Briefly, the ipsilateral striatum on the 21st day post-ICH or from sham-operated rats was harvested and then homogenized in cold radioimmunoprecipitation assay (RIPA) buffer (Cell Signaling Technology) containing 1 mM phenylmethylsulfonyl fluoride (PMSF) and a phosphatase inhibitor cocktail (1:50, Sigma-Aldrich). Simultaneously, the homogenate was centrifuged at 12,000 rpm for 20 min at 4 °C, and the supernatant was collected for protein detection. Twenty-five micrograms of proteins was loaded into each lane and subjected to sodium dodecyl sulfate–polyacrylamide gel electrophoresis (SDS-PAGE) using 4 to 15% Ready Gel (Bio-Rad Laboratories Inc.) at 120 V for 120 to 150 min. Proteins were transferred to polyvinylidene fluoride (PVDF) membranes (Millipore) at 400 mA for 45 min. The PVDF membranes were incubated overnight with primary antibodies at 4 °C, followed by horseradish peroxidase-labeled secondary antibody (Invitrogen) for 1 h at room temperature. All the primary antibodies used are listed in Table S2. Membranes were scanned using the Amersham ImageQuant 800 (Cytiva). The optical densities of all protein bands were analyzed by Image Lab 6.1 software (Bio-Rad Laboratories Inc.).

### Statistical analysis

Statistical analysis was performed by GraphPad Prism software (version 9.5). The quantitative data are presented as the mean ± standard deviation (SD). Unpaired *t* test, 1-way ANOVA, and 2-way ANOVA with repeated measures were used to compare the means of the samples. A value of *P* < 0.05 was considered to indicate a statistically significant difference.

## Results

### Fabrication and characterization of brain ECM hydrogels

**Fig. 2.**
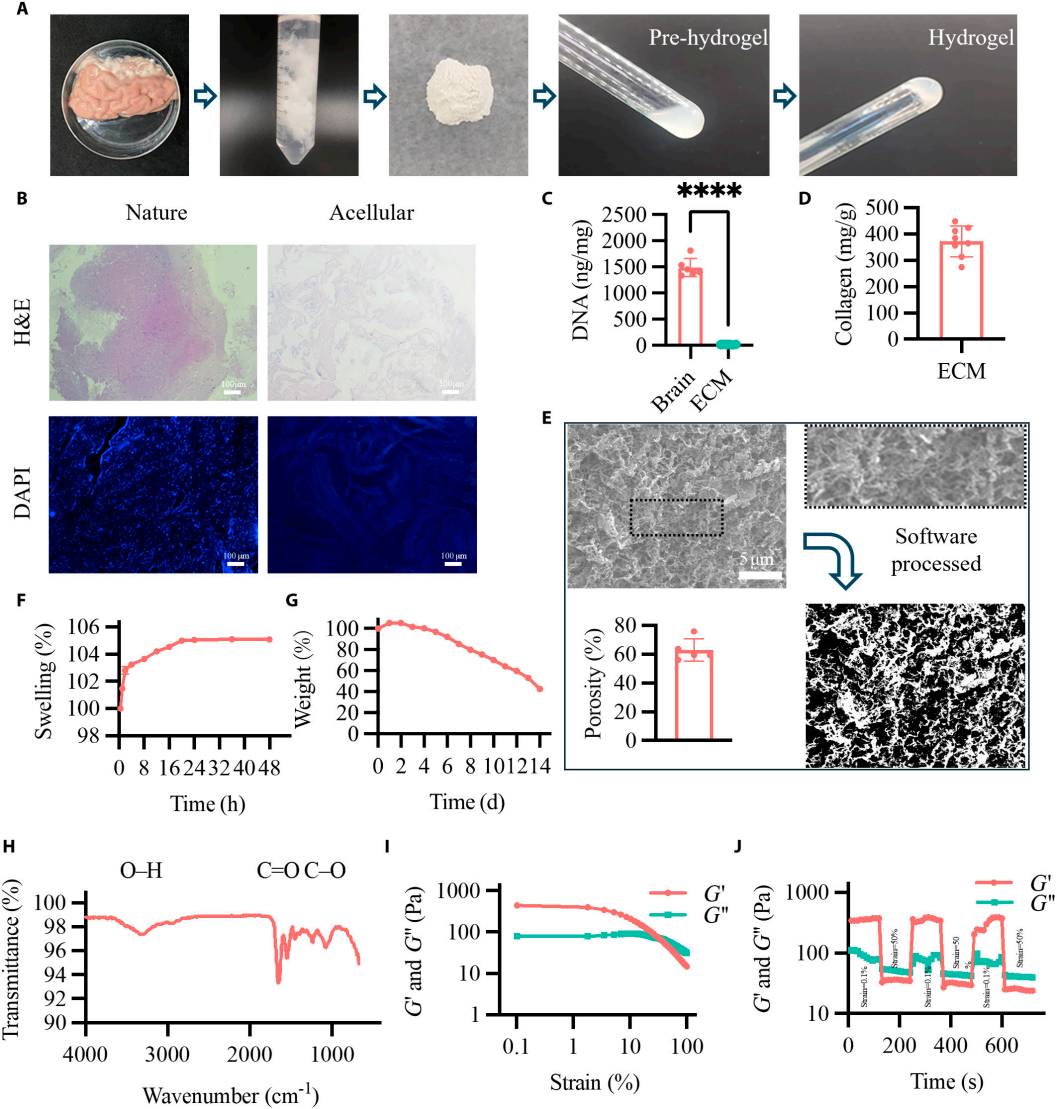
Characterization of brain ECM hydrogel. (A) porcine brain, brain ECM, 300-mesh size of ECM powder, pre-gel ECM solution, and brain ECM hydrogel. (B) H&E staining and DAPI staining of brain and ECM. (C) Quantification of DNA in the brain and ECM. (D) Collagen content of brain ECM hydrogel. (E) Scanning electron micrographs of brain ECM hydrogel. Porosity was calculated using ImageJ software for brain hydrogels. (F and G) Swelling and degradation of brain ECM hydrogel. (H) FT-IR spectroscopy of brain ECM hydrogel. (I and J) Strain-sweep experiments and continuous step strain measurement of brain ECM hydrogel. The results are shown as scatterplots (mean ± SD). ****P < 0.0001.

ECMs were obtained from fully decellularized porcine brains (Fig. 2A). After pepsin digestion, the ECM formed viscous suspensions (pre-gel ECM solution) (Fig. 1A). Hydrogels were prepared after modifying the pH (Fig. 1A). No residual nuclei were visible in the H&E and DAPI images of the brain ECM (Fig. 1B). Quantification of DNA showed that brain ECM scaffolds retained <20 ng /mg (Fig. 1C). The total collagen content of the brain ECM hydrogel was 372.25 ± 58.17 mg/g (Fig. 1D). Scanning electron microscopy (SEM) micrographs revealed a disordered, porous 3D structure of collagen fibrils in the brain ECM hydrogel, with a porosity of 62.93 ± 7.75% (Fig. 1E). The wet weight of the hydrogel increased and reached a saturated state (~105.0%) at 20 h (Fig. 1F) and degraded to 40% at 14 d (Fig. 1G). Fourier transform infrared (FT-IR) spectra of the hydrogels showed that the O–H and C=O stretching bands moved to lower frequencies with greater intensities and bandwidth broadening, indicating molecular interactions such as hydrogen bonding (Fig. 1H). The hydrogels showed viscoelastic-solid behavior. The storage modulus of hydrogels was 442.61 Pa (Fig. 1I). Furthermore, continuous step strain measurements indicate the self-healing property of the hydrogel (Fig. 1J).

### Brain ECM hydrogel promotes motor function recovery after ICH

**Fig. 3.**
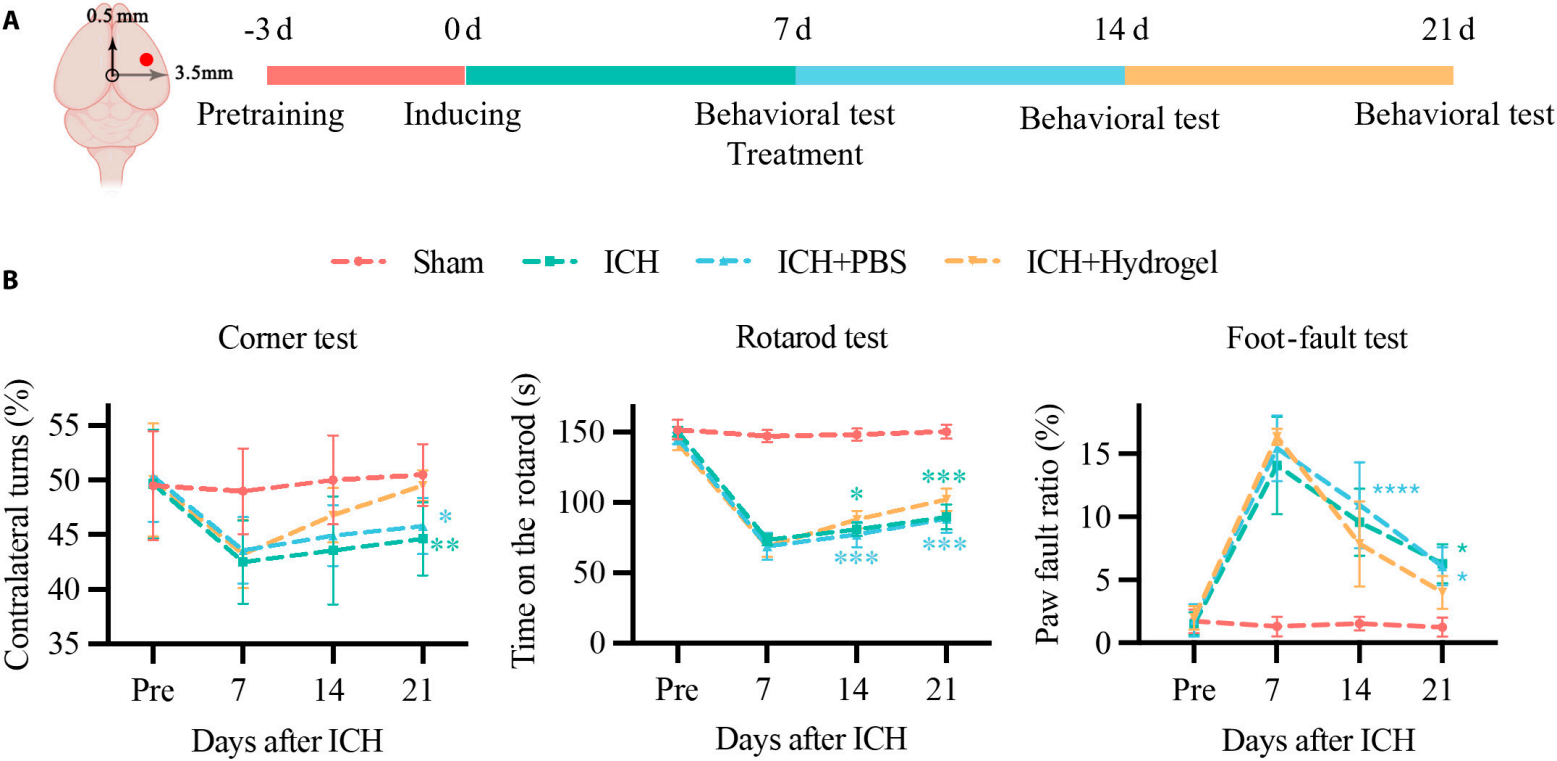
Brain ECM hydrogel promotes motor function recovery after ICH. (A) Flow chart of the animal behavioral test. (B) Behavioral test. *n* = 10 to 14 rats per group. The asterisks indicate a statistically significant difference between this group and the ICH + Hydrogel group. *P < 0.05, **P < 0.01, ***P < 0.001, ****P < 0.0001.

To evaluate the effects of brain ECM hydrogel treatment on motor function, several behavioral tests were performed on days 7, 14, and 21 post-ICH, including the corner test, rotarod test, and foot-fault test (Fig. 3A). The results of the corner test demonstrated that the ICH + Hydrogel group exhibited a significant recovery in contralateral turns compared to both the ICH and ICH + PBS groups (Fig. 3B). Similarly, the rotarod test revealed a significantly increased latency to fall in the ICH + Hydrogel group compared to the ICH and ICH + PBS groups (Fig. 3B). The foot-fault test showed a marked reduction in the error rate of paw placement during grid walking in the ICH + Hydrogel group relative to the ICH and ICH + PBS groups (Fig. 3B). These findings suggest that brain ECM hydrogel treatment effectively ameliorates motor function deficits in rats following ICH.

### Brain ECM hydrogel recruiting endogenous cell migration in the ICH model in vivo

**Fig. 4.**
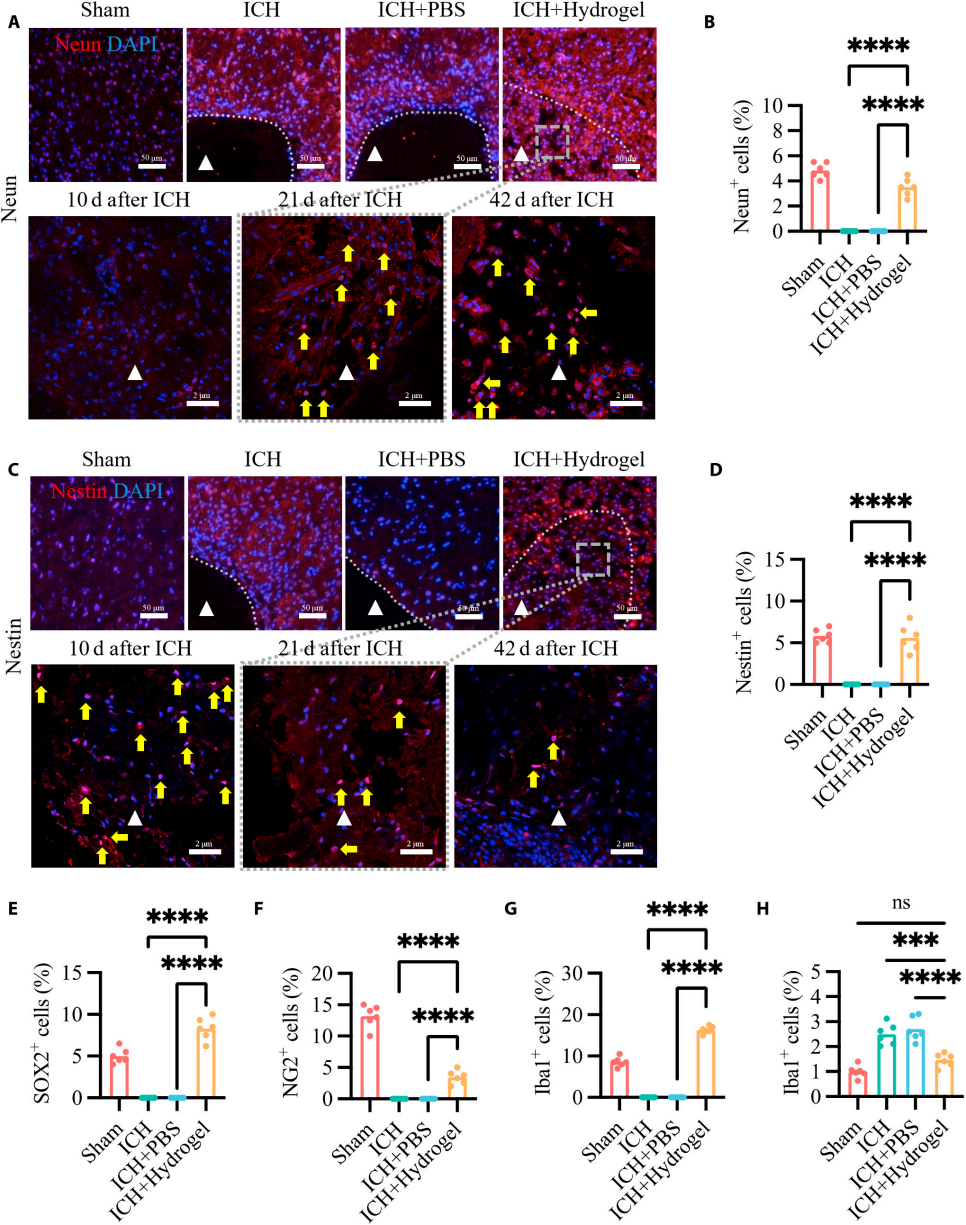
Brain ECM hydrogel promotes endogenous cell migration and reduces inflammation in the peri-cavity area. (A and B) Immunofluorescence staining for NeuN (mature neurons) and semiquantification of NeuN-positive cells in the brain ECM hydrogel (*n* = 6). (C and D) Immunofluorescence staining for Nestin (neural progenitor cells) and semiquantification of Nestin-positive cells in the brain ECM hydrogel (*n* = 6). (E to G) Semiquantification of SOX-2, NG2, and Iba1-positive cells in the brain ECM hydrogel (*n* = 6). (H) Semiquantification of Iba1-positive cells in the peri-cavity area (*n* = 6). The dotted line indicates the interface between the biomaterial and the host brain. Triangles denote the stroke cavity. Yellow arrows highlight positive cells. Data are presented as scatterplots. ns (not significant) > 0.05, ****P < 0.001, ****P < 0.0001.

Immunofluorescence analysis was performed to assess the cellular composition within the brain ECM hydrogel after ICH, focusing on neural cells and neural stem cells. The objective was to evaluate whether the brain ECM hydrogel could promote neurogenesis by facilitating the migration and differentiation of endogenous cells. Infiltration of mature neurons (Fig. 4A and B), new immature neurons (Fig. S1A), neural progenitor cells (Fig. 4C to E), and oligodendrocyte precursor cells (Fig. 4F and Fig. S1B) was observed within the hydrogel at 21 d after ICH induction. To better understand the dynamic changes in cell infiltration, we further analyzed multiple time points (10, 21, and 42 d) after ICH induction. The proportion of NeuN-positive mature neurons increased significantly from the early to the late stage, indicating progressive neuronal maturation (Fig. S1E). In contrast, the proportions of doublecortin (DCX)-positive immature neurons and Nestin-positive progenitor cells gradually decreased over the same period (Fig. S1F and G). This dynamic pattern suggests that neural stem cells within the hydrogel differentiated into mature neurons, supporting the occurrence of neurogenesis.

Although mild infiltration of microglia/macrophages was detected within the hydrogel, the proportion of Iba1-positive cells in the hydrogel was higher than that in the Sham group, indicating an active recruitment of microglia into the hydrogel at 21 d after ICH induction (Fig. 4G and Fig. S1C). However, in the peri-cavity region, the proportion of Iba1-positive cells in the ICH + Hydrogel group was significantly lower than that in the ICH and ICH + PBS groups and was comparable to the Sham group at 21 d after ICH induction (Fig. 4H). This suggests that the brain ECM hydrogel attenuated the microglial/macrophage response in the peri-cavity region, thereby reducing inflammation in the surrounding tissue. Importantly, the infiltration of microglia/macrophages into the hydrogel did not indicate an inflammatory response induced by the hydrogel itself, but rather a controlled recruitment of these cells to support tissue repair and remodeling. The infiltration of these phagocytic cells into the hydrogel occurred via the peri-stroke tissue, crossing the glial scar. These findings indicate that the brain ECM hydrogel within the stroke cavity acts as a scaffold that bridges the gap between brain tissues and promotes the migration of endogenous cells, thereby supporting neurogenesis.

### Brain ECM hydrogel enhances angiogenesis in the peri-cavity area

**Fig. 5.**
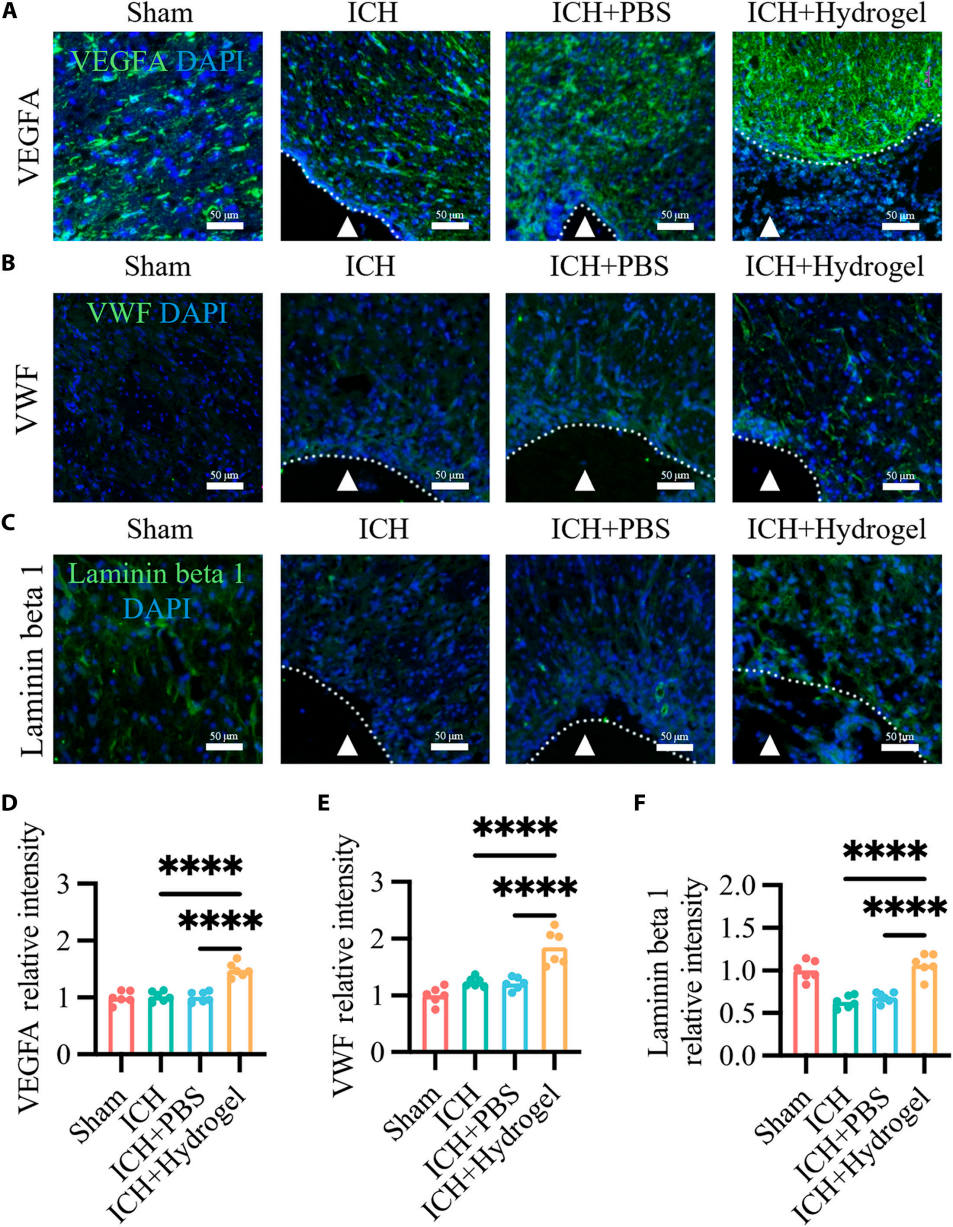
Brain ECM hydrogel enhances angiogenesis in the peri-cavity area. (A to C) Immunofluorescence staining for VEGFA, VWF, and laminin β1 in the peri-cavity area, highlighting angiogenesis. (D to F) Semiquantification of VEGFA, VWF, and laminin β1 fluorescence signals (*n* = 6). The dotted line defines the interface between the biomaterial and the host brain. The triangles indicate the stroke cavity. The results are shown as scatterplots. ****P < 0.0001.

To investigate the effects of the hydrogel on angiogenesis in the peri-cavity region, we examined the expression of key angiogenic markers. Immunofluorescence staining for vascular endothelial growth factor A (VEGFA) (Fig. 5A and D), von Willebrand factor (VWF) (Fig. 5B and E), and laminin β1 (Fig. 5C and F) at 21st day post-ICH revealed that hydrogel treatment significantly promoted neovascularization in the peri-cavity area.

### Brain ECM hydrogel attenuated neuronal cell injury in response to hemin in vitro

To evaluate the protective effects of brain ECM hydrogel on neuronal cells in an in vitro ICH model, N2a and SH-SY5Y cells were exposed to 10 μM hemin for 24 h to induce cell injury, followed by treatment with sterilized brain ECM hydrogel (0.1 g/ml) for an additional 24 h. The concentration of 10 μM hemin was selected based on its established use in modeling ICH-induced neuronal injury in both N2a and SH-SY5Y cells [28,29]. The concentration of brain ECM hydrogel (0.1 g/ml) was determined through a concentration gradient experiment using Cell Counting Kit-8 (CCK-8) assays. The brain ECM hydrogel significantly promoted cell proliferation, and the concentration of 0.1 g/ml was selected for subsequent experiments (Fig. S2A). The effects of this treatment on apoptosis, proliferation, and cell viability were then assessed.

**Fig. 6.**
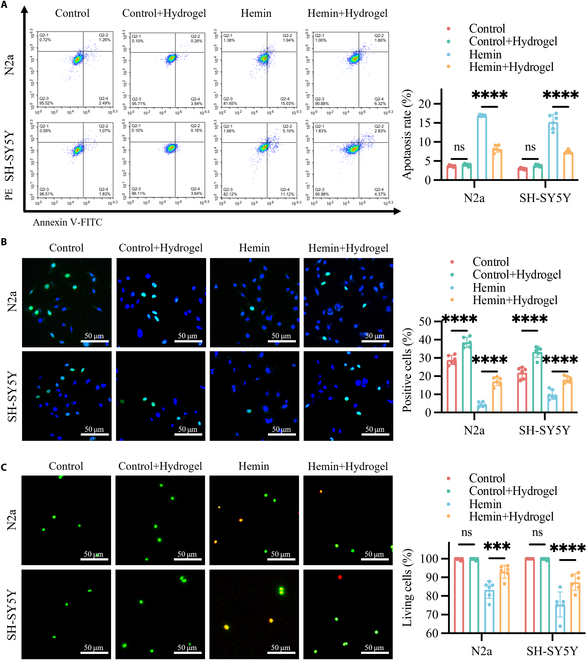
Brain ECM hydrogel attenuated neuronal cell injury in response to hemin in vitro. (A) Apoptosis of N2a and SH-SY5Y cells in the control, control + hydrogel, hemin, and hemin + hydrogel groups, assessed by flow cytometry. (B) Proliferation of N2a and SH-SY5Y cells in the control, control + hydrogel, hemin, and hemin + hydrogel groups, measured by EdU incorporation. (C) Cell viability of N2a and SH-SY5Y cells in the control, control + hydrogel, hemin, and hemin + hydrogel groups, evaluated by Live/Dead assay. Results are presented as scatterplots (mean ± SD). *n* = 6. ns > 0.05, ***P < 0.001, ****P < 0.0001.

Flow cytometry analysis revealed that apoptosis in hemin-treated cells was significantly reduced following brain ECM hydrogel treatment (Fig. 6A). Importantly, the control + hydrogel group exhibited no significant difference in apoptosis compared to the control group, indicating that the hydrogel itself does not induce apoptosis under normal physiological conditions. In terms of cell proliferation, both 5-ethynyl-2′-deoxyuridine (EdU) incorporation and CCK-8 assays demonstrated a marked enhancement in the proliferative capacity of hemin + hydrogel-treated cells compared to the hemin-induced group (Fig. 6B and Fig. S2B). Similarly, the control + hydrogel group displayed a significant increase in proliferation relative to the control group, suggesting that the hydrogel can promote cell proliferation even in the absence of pathological stress. Furthermore, Live/Dead staining indicated a significant improvement in the viability of hemin + hydrogel-treated cells compared to the hemin-induced group (Fig. 6C). Notably, the control + hydrogel group showed no significant difference in cell viability compared to the control group, confirming that the hydrogel does not adversely affect cell survival under normal conditions.

### Brain ECM hydrogel reverse transcriptomic changes in vitro

**Fig. 7.**
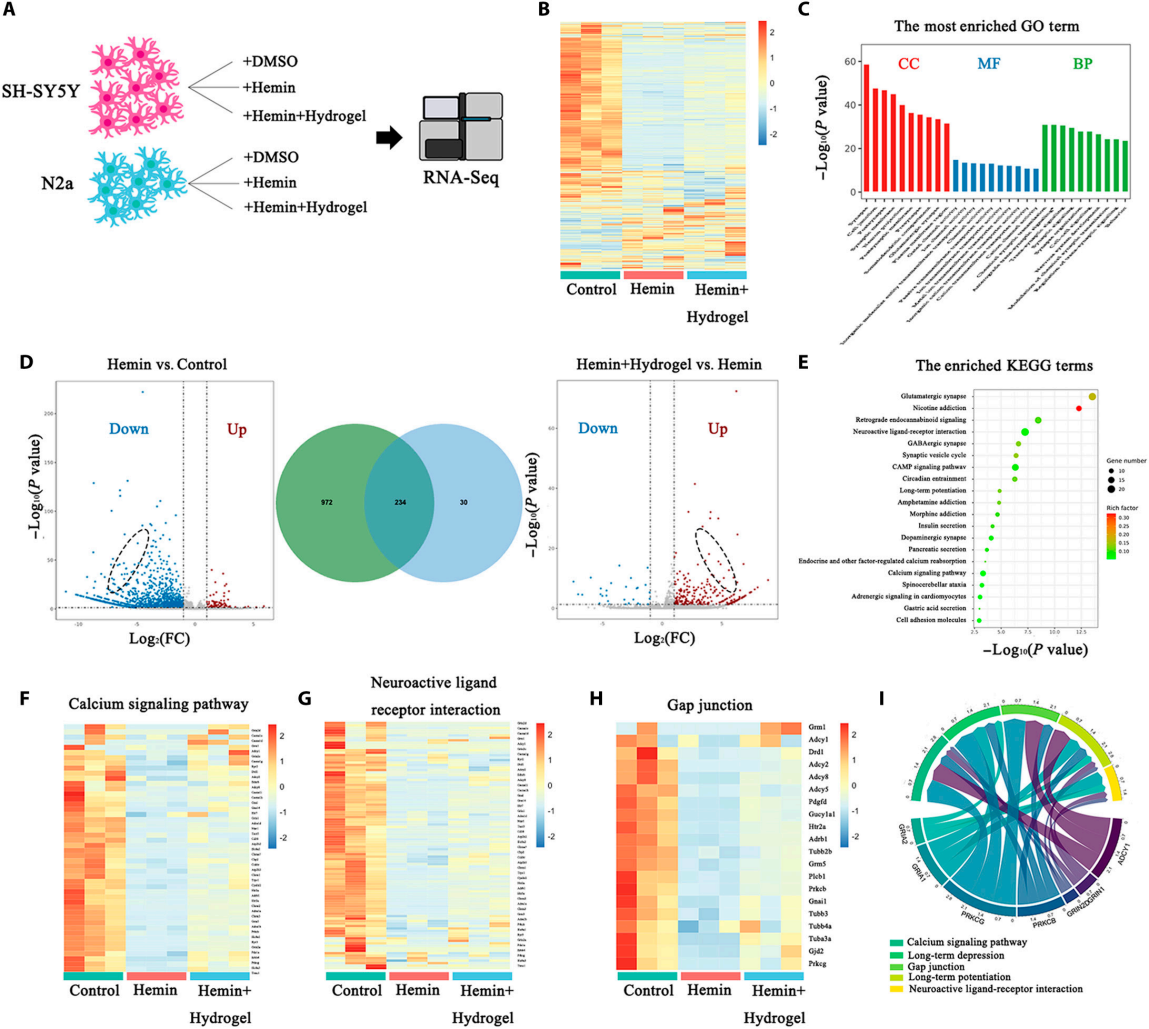
RNA sequencing analysis of differentially expressed genes. (A) Schematic of hydrogel treatment RNA sequencing (RNA-Seq) experimental design. SH-SY5Y and N2a cells were treated with either dimethyl sulfoxide (DMSO), hemin, or hemin + hydrogel. Cells were then subjected to RNA sequencing. (B) Heatmaps of differentially expressed mRNAs from the RNA sequencing analysis of the control group, the hemin group, and the hemin + hydrogel group in SH-SY5Y cells. (C) GO enrichment analysis in the hemin + hydrogel group compared with the hemin group. (D) The number of genes with lower expression (≥2-fold difference) in the hemin group compared with the control group and higher expression in the hemin + hydrogel group compared with the hemin group. (E) Top 20 significant KEGG pathways in the hemin + hydrogel group compared with the hemin group. (F to H) Heatmaps show the relative expression of genes related to calcium signaling pathway, neuroactive ligand–receptor interaction, and gap junction in the control group, the hemin group, and the hemin + hydrogel group. (I) GOChord plot of the GO enrichment analysis in the hemin + hydrogel group compared with the hemin group.

To reveal the crucial targets for the protective effect of brain ECM hydrogel in the hemin-induced ICH model, RNA sequencing analysis was conducted among the control group, hemin group, and hemin + hydrogel group in SH-SY5Y and N2a cells (Fig. 7A). While the heatmap indicates that the hydrogel-treated group displayed a gene expression profile with certain similarities to the hemin group, this observation reflects a partial reversal of hemin-induced transcriptomic changes rather than a complete restoration to the control state. This is consistent with the hydrogel’s neuroprotective role in mitigating hemin-induced injury. First, differential expression genes (DEGs) between the hemin group and the control group were screened in SH-SY5Y cells (Fig. 7B). The results showed that 83 differentially up-regulated genes and 1,206 differentially down-regulated genes were obtained. Then, DEGs between the hemin + hydrogel group and the hemin group were screened, and 264 differentially up-regulated genes and 29 differentially down-regulated genes were found (Fig. 7D). Notably, 234 genes were identified both in the down-regulated genes of the hemin group versus the control group and in the up-regulated genes of the hemin + hydrogel group versus the hemin group (Fig. 7D).

The Gene Ontology (GO) enrichment results indicated that the biological process of brain ECM hydrogel neuroprotection was related to chemical synaptic transmission, synaptic signaling, cell–cell signaling, and nervous system development. Furthermore, the significant signaling pathways of Kyoto Encyclopedia of Genes and Genomes (KEGG) enrichment analysis were mainly involved in the glutamatergic synapse, neuroactive ligand–receptor interaction, γ-aminobutyric acid-mediated (GABAergic) synapse, and synaptic vesicle cycle (Fig. 7C). These pathways are fundamental to neuronal function and survival, and their modulation by the hydrogel suggests a neuroprotective mechanism that counteracts hemin-induced injury. While these pathways are not exclusively associated with neuroprotection or neurogenesis, their regulation by the hydrogel supports its role in mitigating neuronal damage and promoting functional recovery.

Then, we conducted gene set enrichment analysis (GSEA) to determine these affected pathways. Calcium signaling pathway, neuroactive ligand–receptor interaction, and gap junction were down-regulated in the hemin group and up-regulated in the hemin + hydrogel group (Fig. 4F to H). To identify the key upstream DEGs related to these biological processes, we used GOChord plots. The data indicated that 7 genes were related to more than 5 biological processes and had a log_2_ fold change > 0 (positive effect): adenylyl cyclase 1 (ADCY1), glutamate ionotropic receptor NMDA type subunit 1 (GRIN1), glutamate ionotropic receptor NMDA type subunit 2D (GRIN2D), protein kinase C β (PRKCB), protein kinase C γ (PRKCG), glutamate ionotropic receptor AMPA type subunit 1 (GRIA1), and glutamate ionotropic receptor AMPA type subunit 2 (GRIA2) (Fig. 7I).

To further illustrate the RNA sequencing results, supplementary figures provide detailed insights into differentially expressed genes (DEGs) across groups, including heatmaps of mRNA expression in N2a cells (Fig. S3), comparative analyses of gene expression changes (Fig. S4A and B), and enrichment analyses of biological processes and KEGG pathways (Fig. S5A and B). Additionally, Figs. S6 and S7 illustrate the relative expression of key signaling pathways among the DEGs in N2a cells. Figure S8 shows the protein–protein interaction networks in both N2a and SH-SY5Y cells. The partial reversal of hemin-induced transcriptomic changes by the hydrogel, as observed in the heatmap and DEG analysis, underscores its neuroprotective role. While the hydrogel does not fully restore the cells to the control state, its modulation of fundamental neuronal pathways supports its potential as a therapeutic intervention for ICH-induced neuronal injury.

### Brain ECM hydrogel recruiting endogenous cell migration via the Slit2-Robo1 signaling pathway

**Fig. 8.**
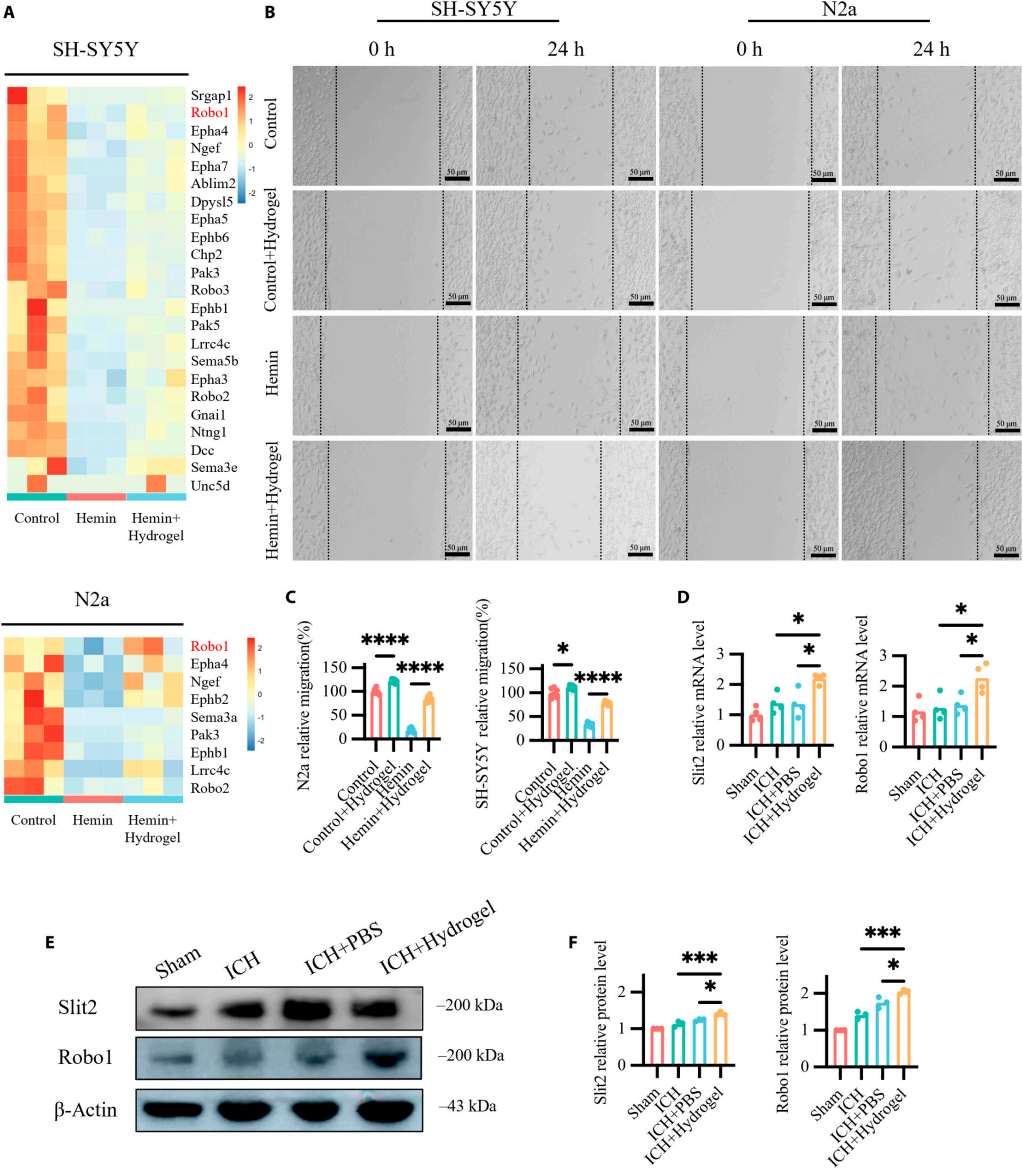
Brain ECM hydrogel recruiting endogenous cell migration via the Slit2-Robo1 signaling pathway. (A) Heatmaps show the relative expression of genes related to axon guidance in the control group, the hemin group, and the hemin + hydrogel group. (B and C) Cell migration of neurons in the control, control + hydrogel, hemin, and hemin + hydrogel groups, as assessed by scratch assay. Representative images and quantifications are shown. (D) The mRNA levels of Slit2 and Robo1 were detected by RT-qPCR (*n* = 4/group). (E) Representative Western blot images showing the protein levels of Slit2 and Robo1 in the ICH rat’s brains. (F) Quantification analysis of the levels of Slit2 and Robo1 by Western blot (*n* = 3/group). The results are shown as scatterplots. *P < 0.05, ***P < 0.001, ****P < 0.0001.

RNA sequencing analysis revealed that the hydrogel group exhibited a gene expression profile partially resembling the hemin group (Fig. 8A), likely reflecting the shared inflammatory microenvironment induced by hemin. Notably, Robo1, a key regulator of neural cell migration, was significantly up-regulated in the hydrogel group (Fig. 8A). To explore the mechanisms of hydrogel-mediated cell recruitment, we conducted in vitro and in vivo experiments. A scratch assay demonstrated that hemin-treated cells exhibited impaired migration compared to hydrogel-treated cells (Fig. 8B and C), while the control + hydrogel group displayed enhanced migration relative to the control group, indicating the hydrogel’s intrinsic pro-migratory properties. qPCR and Western blot analyses revealed up-regulation of Slit2 and Robo1 in the hydrogel-treated group at 21 d after ICH induction (Fig. 8D to F), and immunofluorescence staining confirmed higher Robo1 expression in the peri-cavity region of the ICH + Hydrogel group compared to controls (Fig. S9). Collectively, these findings suggest that the brain ECM hydrogel recruits endogenous cell migration by activating the Slit2 and Robo1 signaling pathways.

## Discussion

For the treatment of ICH, aside from surgical intervention during the acute phase, there are currently no clinically validated techniques available [30]. Most preclinical studies focus on the acute treatment window following ICH. However, research on long-term repair post-ICH remains lacking. Neurorepair, including angiogenesis and neurogenesis, is currently believed to begin from the subacute to the chronic phase following acute brain injury and to persist for several weeks or months. However, the exact timeline and mechanisms of regenerative events after ICH remain unclear [31,32]. In this study, we found that brain ECM hydrogel improved neurological function in ICH rats, promoted endogenous cell migration, and enhanced both the proliferation and migration of cells in vitro.

Brain ECM provides a favorable microenvironment that supports the adhesion, proliferation, and differentiation of neural cells, including neurons and glial cells. Previous reports have confirmed that the implantation of ECM can promote improvements in neurobehavioral functions in animal models of traumatic brain injury [17,33,34]. However, there are limitations to the practical application of ECM. The degradation rate and release characteristics of natural ECM are difficult to precisely control, which may be one of the reasons for its suboptimal effectiveness and safety in specific applications. The hydrogel form of ECM expands its potential. The physical and chemical properties of hydrogels, such as stiffness, degradation rate, and pore structure, can be regulated by adjusting the composition and preparation conditions, significantly enhancing the controllability of ECM use. ECM hydrogels can remain in the tissue cavity for extended periods post-implantation, showing only moderate biodegradation within 12 weeks [35]. In this study, the hydrogel demonstrated substantial retention both in vitro and in vivo over a 5-week observation period. The storage modulus of 10 mg/ml brain ECM hydrogel is similar to that of brain tissue [36]. Brain ECM hydrogel does not cause mass effect leading to brain herniation. Additionally, the hydrogel can be injected into the deep brain through a needle to fill irregularly shaped cavities formed after ICH. The hydrogel provides a 3D structure within the lesion, mimicking the microenvironment of brain tissue in vivo, supporting the spatial distribution and connection of neural cells, and better promoting the functional recovery.

The role of the glial scar remains controversial. Glial scars and myelin debris in the surrounding environment may be significant inhibitors of axonal regeneration, but their role and switching mechanisms in early neural repair post-ICH remain unclear. Cell migration into the ECM hydrogel is a critical and necessary event for tissue regeneration. Previous studies have shown that injury sites sealed by glial scars accumulate a variety of nonneural cell types with diverse origins and significant heterogeneity. After the application of ECM hydrogel, the expression of Nestin, SOX-2, DCX, NG2, NeuN, and IBA-1 is observed within the hydrogel and in the brain tissue surrounding the cavity. Microglia/macrophages infiltrate the ECM hydrogel, initiating a process of constructive remodeling. During this process, these cells may release molecular cues that establish migratory pathways, thereby facilitating the recruitment and gradual repopulation of host tissue cells within the material [37]. Compared to other studies [20,21], our research indicates that brain ECM hydrogel can better attract neural progenitor cells, oligodendrocyte precursor cells, and phagocytes to migrate, suggesting that infiltration of endogenous neural cells can be observed even before the formation of glial scars. Tuszynski’s team discovered that some axons can regenerate into scar tissue coexpressing NG2 [a member of chondroitin sulfate proteoglycans (CSPGs)], L1, and laminin or NCAM (the latter 3 being cell adhesion molecules considered to be axonal growth-promoting factors), implying that the balance between inhibitory and promoting factors at the injury site may regulate the regeneration of certain types of neural axons. Over the past decade, there has been deeper research into the heterogeneity and complexity of glial cell physiological functions in the central nervous system. Glial cells exhibit characteristics different from their normal state upon injury. Attempting to measure their role in injury repair by simply eliminating a specific cell type may significantly differ from reality. The function and role of the glial scar in neural regeneration or repair likely require more complex, in-depth research and comprehensive considerations.

The ECM contains various bioactive molecules (such as growth factors and cell adhesion molecules) that can regulate cellular behavior. Additionally, certain components of the ECM possess neuroprotective properties, promoting neuronal survival and axonal growth. Our study found that brain ECM hydrogel can protect neuronal cells following ICH. For example, nerve growth factor (NGF) binds to receptors on the surface of neurons (primarily TrkA and p75NT receptors), activating a series of signaling pathways that promote the expression of proliferation and survival genes, such as Bcl-2 and Bcl-x, further enhancing neuronal survival. Brain-derived neurotrophic factor (BDNF), by binding to the TrkB receptor, activates multiple signaling pathways, inhibiting the activity of the apoptotic protein Caspase-3 and enhancing anti-apoptotic capacity. Various neurotrophic factors also regulate microtubule-associated proteins, enhancing microtubule stability and dynamics, and promoting axonal extension. Furthermore, the hydrogel form of ECM mimics the 3D structure of the natural ECM, guiding axonal regeneration and providing a supportive microenvironment for neural cells, aiding the restoration of functional connections in damaged nerve fibers. The specific components and 3D structure of brain ECM hydrogel synergize in the process of neural repair, comprehensively regulating neuronal survival, cytoskeleton remodeling, axonal guidance, and extension through different receptors and signaling pathways, providing overall support. Additionally, our study found that brain ECM hydrogel increased the expression of VWF, laminin β1, and VEGFA in the peri-cavity area. The elevated levels of these biomarkers are indicative of beneficial regeneration and play critical roles in the repair process. Specifically, VEGFA promotes angiogenesis and neuroprotection [38,39]. The increased expression of VEGFA in the peri-cavity area may be attributed to enhanced secretion by neural cells, glial cells, and endothelial cells, which could include both cells within the ECM hydrogel and those in the surrounding peri-cavity region [39]. This enhanced secretion may result from both the improved microenvironment created by the brain ECM hydrogel and the increased activity of these cells in response to hemorrhagic injury. VWF, on the other hand, promotes vascular maturation by facilitating the coverage of the endothelial basement membrane with smooth muscle cells [40], thereby stabilizing newly formed blood vessels. Laminin β1, a key component of the ECM, regulates angiogenesis and supports the structural integrity of the vascular network [41]. Together, these mechanisms synergistically enhance vascularization, as evidenced by the increased formation of new blood vessels at the lesion edges in the ECM hydrogel group compared to the control. This improved vascularization restores blood supply, providing essential nutrients and oxygen for tissue repair, which may contribute to the recovery of neural function.

Moreover, we observed that brain ECM hydrogel reduced the microglial/macrophage response at the lesion edges, decreasing the inflammatory response around the stroke cavity. This suggests that brain ECM hydrogel may reduce post-neurodamage inflammation by releasing anti-inflammatory factors or modulating the immune response, thereby promoting the repair process.

In transcriptomic analyses, we found that genes related to neuronal growth and development were down-regulated in neurons within the in vitro ICH model. GSEA revealed that affected pathways, including neuroactive ligand–receptor interaction, axon guidance, and gap junction, were down-regulated. Previous studies have shown that the Slit2-Robo1 pathway contributes to cell migration and supports neurological recovery after stroke [42,43]. Our findings suggest that brain ECM hydrogel may promote cell migration and enhance neural recovery after ICH by mediating the Slit2-Robo1 pathway.

The choice of porcine-derived brain ECM hydrogel in this study was driven by its tissue specificity, biodegradability, and biocompatibility, with porcine tissue sharing key similarities with human brain ECM, such as collagen and laminin, which are crucial for promoting cell migration and tissue repair. Porcine ECM has been widely used in preclinical studies for treating central nervous system injuries, including stroke and traumatic brain injury, and offers a consistent and ethically acceptable source of material for such applications [44,45]. However, while porcine ECM presents significant advantages, its use in human therapy raises concerns regarding immune rejection due to potential residual xenogenic components, as well as variations in ECM composition that can arise from differences in donor age, health status, and genetic background. These factors may influence the mechanical properties and biological activity of the ECM hydrogel. Ideally, human-derived ECM would offer better immunological compatibility and relevance to human tissue, but ethical and logistical challenges make this option less feasible. Therefore, porcine ECM remains a practical alternative, although ongoing advancements in decellularization techniques and a deeper understanding of the impact of donor variability are needed to optimize ECM-based therapies and reduce immunogenic risks.

In summary, we have successfully developed a brain ECM hydrogel with notable biodegradability and biocompatibility. In an ICH rat model, the hydrogel facilitated neural recovery by enhancing cell recruitment, angiogenesis, and neurogenesis while mitigating inflammation. Furthermore, it promoted cell proliferation and migration, inhibited apoptosis, and modulated transcriptomic changes in vitro. The hydrogel also appears to support neuronal migration and functional recovery through the Slit2-Robo1 signaling pathway. These bioactive properties underscore the potential of brain ECM hydrogel for advancing neural repair strategies following ICH.

## Data Availability

The datasets used and/or analyzed during the current study are available from the corresponding author on reasonable request.
