# Supplementary material for: Injectable Brain Extracellular Matrix Hydrogels Enhance Neuronal Migration and Functional Recovery After Intracerebral Hemorrhage
Source: Biomater Res. 2025 Apr 22;29:0192. doi: 10.34133/bmr.0192 (PMC12012376; doi:10.34133/bmr.0192)
Supplement: Supplementary 1 — Materials and Methods Figs. S1 to S9 Tables S1 and S2 [file bmr.0192.f1.doc]

Supporting Information For

Injectable Brain Extracellular Matrix Hydrogels Enhance Neuronal Migration and Functional Recovery after Intracerebral Hemorrhage

**Authors**

Jiajie Xia^1,2#^, Xinjie Gao^3#^, Jun Yao^4#^, Yuchao Fei^3^, Dagang Song^2^, Zhiwei Gu^2^, Gang Zheng^2^, Yuxiang Gu^1,3*^, Chuanjian Tu^5*^

**Affiliations**

^1^ Department of Neurosurgery, Neurosurgery Research Institute, The First Affiliated Hospital, Fujian Medical University, Fuzhou, Fujian, 350005, China.

^2^ Department of Neurosurgery, Shaoxing Central Hospital, The Central Affiliated Hospital, Shaoxing University, Shaoxing, Zhejiang, 312030, China.

^3^ Department of Neurosurgery of Huashan Hospital, State key Laboratory of Medical Neurobiology, MOE Frontiers Center for Brain Science, and Institutes of Brain Science, Fudan University, Shanghai, 200000, China.

^4^ Department of Orthopedic Surgery, Shaoxing Central Hospital, The Central Affiliated Hospital, Shaoxing University, Shaoxing, Zhejiang, 312030, China.

^5^ Department of Neurosurgery, Shaoxing Central Hospital, China Medical University, Shaoxing, Zhejiang, 312030, China.

^#^ These authors contribute equally.

^*^ Indicates the corresponding author

^*^ Corresponding author: Yuxiang Gu, Chuanjian Tu

E-mail address: guyuxiang1972@126.com (Yuxiang Gu), tuchj@usx.edu.cn (Chuanjian Tu)

**SUPPLEMENTARY MATERIALS**

**Supplementary materials and methods**

*Scanning electron microscopy*

The morphological properties of brain ECM hydrogel scaffolds were examined by SEM (JSM-6360LV, JEOL). To prepare samples for imaging, the scaffold was supercritical CO2 dried, stuck to conductive electrical tape, and coated with gold for 120 s.

*Characterizations and rheological measurements of hydrogels*

The chemical structures of the hydrogels were analyzed with an FT-IR spectroscopy (Nicolet Nexus, Thermo Scientific, USA) using the attenuated total reflection (ATR) technique with a wavenumber range of 675–4000 cm^-1^.

The MCR302 Rheometer with a tribological cell (Anton Paar France S.A.S.) is equipment adapted for research work. The hydrogels were freshly prepared and added to the plate immediately. The environmental temperature was controlled at 37 °C. The strain-sweep experiments were carried out from 0.1% to 100% dynamic strain amplitudes for the hydrogels at 10 rad/s frequency. In addition, the self-healing properties of hydrogels were evaluated by rheological analysis. Continuous step strain measurement with 10 rad/s frequency was processed for hydrogels at alternate 1% and 50% dynamic strains for 120 s at each step.

*Collagen quantification via hydroxyproline (HYP) content assay*

Collagen content in tissue samples was quantified by measuring hydroxyproline (HYP) levels, as hydroxyproline is a major amino acid component of collagen. The Hydroxyproline Content Assay Kit (Sangon Biotech) was used according to the manufacturer's instructions. Tissue samples were first subjected to acid hydrolysis to release free hydroxyproline. The hydrolyzed samples were then oxidized with Chloramine T, and the resulting oxidized products were reacted with p-Dimethylaminobenzaldehyde (DMAB). This reaction formed a red-colored compound, which exhibited a characteristic absorbance peak at 560 nm. The absorbance at 560 nm was measured using a microplate reader, and the hydroxyproline content was calculated by comparing the sample’s absorbance to a standard curve generated from known concentrations of hydroxyproline. The hydroxyproline content was then used to calculate the collagen content in the tissue samples.

*Swelling and degradation study*

The hydrogels were freshly prepared and incubated in PBS at 37 °C. The swelling property was determined by examining the wet weight of each hydrogel at a certain time point. The degradation profile was determined by examining the remaining dry weight of each hydrogel. The dry weight of each sample was measured at a certain time point. Three samples for each group (n = 3) were measured in the swelling and degradation study.

*Cell cultures*

N2a mouse neuroblastoma cells (Cat No. CCL-131) and SH-SY5Y human neuroblastoma cells (Cat No. CRL-2266) were obtained from the American Type Culture Collection (ATCC). Both cell lines were cultured in Dulbecco's modified Eagle's medium (DMEM) (Procell, #PM150210) supplemented with 10% fetal bovine serum (FBS) (BioInd, #04-001-1ACS). All media were further supplementaed with 1% penicillin streptomycin (Cytiva, #SV30010) to prevent contamination. Cells were incubated in a humidified incubator containing 5% CO2 at 37°C.

To establish an in vitro intracerebral hemorrhage (ICH) model, N2a and SH-SY5Y cells were incubated with 10 μM hemin (Aladdin) for 24 hours, as described in previous studies. Following hemin exposure, cells were treated with sterilized brain ECM hydrogel at a concentration of 10 mg/mL for an additional 24 hours. The experimental design included four groups: (1) control group (untreated cells), (2) control + hydrogel group (cells treated with brain ECM hydrogel alone), (3) hemin group (cells exposed to hemin alone), and (4) hemin + hydrogel group (cells exposed to hemin followed by treatment with brain ECM hydrogel).

*RNA-seq*

Total RNA was extracted from SH-SY5Y and N2a cells, which were divided into three experimental groups: control, hemin, and hemin + hydrogel, with three biological replicates per group. RNA extraction was performed using TRIzol reagent (Thermo Fisher Scientific) according to the manufacturer’s protocol. The RNA concentration was measured based on the A260/A280 absorbance ratio using a Nanodrop ND-2000 system (Thermo Scientific, USA), and RNA integrity (RIN value) was assessed using an Agilent Bioanalyzer 4150 system (Agilent Technologies, CA, USA). Only RNA samples meeting quality control criteria were used for library preparation.

Paired-end RNA-seq libraries were constructed using the ABclonal mRNA-seq Lib Prep Kit (ABclonal, China) following the manufacturer’s instructions. Briefly, mRNA was purified from 1 μg of total RNA using oligo(dT) magnetic beads, followed by fragmentation using divalent cations at elevated temperatures in the ABclonal First Strand Synthesis Reaction Buffer. First-strand cDNA was synthesized using random hexamer primers and Reverse Transcriptase (RNase H) with fragmented mRNA as templates, followed by second-strand cDNA synthesis using DNA Polymerase I, RNase H, buffer, and dNTPs. The double-stranded cDNA fragments were adapter-ligated for paired-end library preparation, followed by PCR amplification. PCR products were purified using the AMPure XP system, and the quality of the libraries was assessed using an Agilent Bioanalyzer 4150 system. Sequencing was performed on an Illumina NovaSeq 6000 platform, generating 150 bp paired-end reads. The sequencing data were processed and analyzed using an in-house bioinformatics pipeline developed by Shanghai Applied Protein Technology. Key analytical steps and tools included the following:

Data Preprocessing: Raw sequencing reads (FASTQ format) were processed using in-house Perl scripts. Adapter sequences were removed, and low-quality reads (reads with >60% of bases having a quality score ≤25) and those with N (undefined base) content >5% were filtered out. Clean reads were obtained for subsequent analysis.

Alignment: Clean reads were aligned to the reference genome in orientation mode using HISAT2, producing mapped reads.

Gene Expression Quantification: FeatureCounts was used to count the number of reads mapped to each gene. Gene expression levels were calculated as fragments per kilobase of transcript per million mapped reads (FPKM), normalized based on gene length and mapped read counts.

Differential Expression Analysis: Differential expression analysis was performed using DESeq2. Genes with |log2FoldChange| > 1 and adjusted P-value (Padj) < 0.05 were defined as significantly differentially expressed genes (DEGs).

Functional Enrichment Analysis: GO function enrichment, KEGG pathway enrichment analysis, and gene set enrichment analysis (GSEA) were conducted to explore the biological significance of the DEGs and to identify functional differences among experimental groups. These analyses were performed using the clusterProfiler R package. Enrichment was considered statistically significant when P < 0.05.

*Cell proliferation assay*

Cell proliferation was assessed using both the Cell Counting Kit-8 (CCK-8) (GlpBio) and the EdU Cell Proliferation Kit with Alexa Fluor 488 (Beyotime) according to the manufacturer’s protocols. For the CCK-8 assay, cells were seeded into 96-well plates at a density of 5 × 10³ cells per well. After treatment, 10 μL of CCK-8 solution was added to each well, and the cells were incubated for 2 hours at 37°C. The absorbance at 450 nm was measured using a microplate reader (BioTek, USA). For the EdU assay, cells were incubated with EdU solution (10 μM) for 2 hours, followed by fixation in 4% paraformaldehyde. EdU incorporation was detected using a fluorescence microscope after the cells were treated with the Alexa Fluor 488 conjugate. The percentage of proliferating cells was calculated based on the ratio of EdU-positive cells to total DAPI-stained cells.

*Cell apoptosis assay*

To assess cell apoptosis, the Annexin V Apoptosis Plate Assay Kit (Dojindo) was used in accordance with the manufacturer’s instructions. Following treatment, cells were collected, washed with cold PBS, and resuspended in 1x binding buffer. Annexin V-FITC and propidium iodide (PI) were added to the cell suspension, and the cells were incubated at room temperature for 15 minutes in the dark. Apoptotic cells were analyzed by flow cytometry using a Novocyte Flow Cytometer (ACEA Bioscience, USA). Early apoptotic cells were defined as Annexin V-positive/PI-negative, while late apoptotic or necrotic cells were defined as Annexin V-positive/PI-positive. Data analysis was performed using FlowJo 10.0 software (FlowJo LLC, USA), with the percentage of apoptotic cells calculated relative to the total cell population.

*Cell viability assay*

Cell viability was assessed using the Live/Dead Cell Staining Kit (BioVision) following the manufacturer’s instructions. Briefly, cells were incubated with a mixture of calcein-AM (which stains live cells) and propidium iodide (PI) (which stains dead cells) for 30 minutes at 37°C. After incubation, cells were washed with PBS, and images were captured using a fluorescence microscope (Nikon, Japan). Live cells were stained green, while dead cells were stained red. The proportion of viable cells was calculated by counting the number of green-stained cells relative to the total cell population, including both live and dead cells.

*Cell migration assay*

Cell migration was evaluated using a scratch wound healing assay. Cells were seeded into 6-well plates at a density of 1 × 10⁶ cells per well and grown to confluence. A uniform wound was created by gently scraping the cell monolayer with a P200 pipette tip. The plates were washed with PBS to remove detached cells, and the cells were cultured in serum-free medium for the duration of the experiment. The closure of the wound was monitored at 0 and 24 hours by capturing images using an inverted fluorescence microscope (Nikon, Japan). The migration distance was measured, and the percentage of wound closure was calculated by comparing the area of the wound at the two time points.

**Supplementary figures**

Figure S1. (A, G) Immunofluorescence staining for DCX (a marker of newly generated immature neurons) and semi-quantification of DCX-positive cells in the ICH + Hydrogel group (n = 6). (B-D) Immunofluorescence staining for NG2 (a marker of oligodendrocyte precursor cells) and Iba1 (a marker of microglia/macrophages). (E-F) Semi-quantification of NeuN (a marker of mature neurons) and Nestin (a marker of neural stem/progenitor cells)-positive cells in the ICH + Hydrogel group (n = 6). The dotted line indicates the interface between the biomaterial and the host brain. Triangles denote the stroke cavity. Yellow arrows highlight positive cells. Data are presented as scatter plots. **P* < 0.05, *****P* < 0.0001.


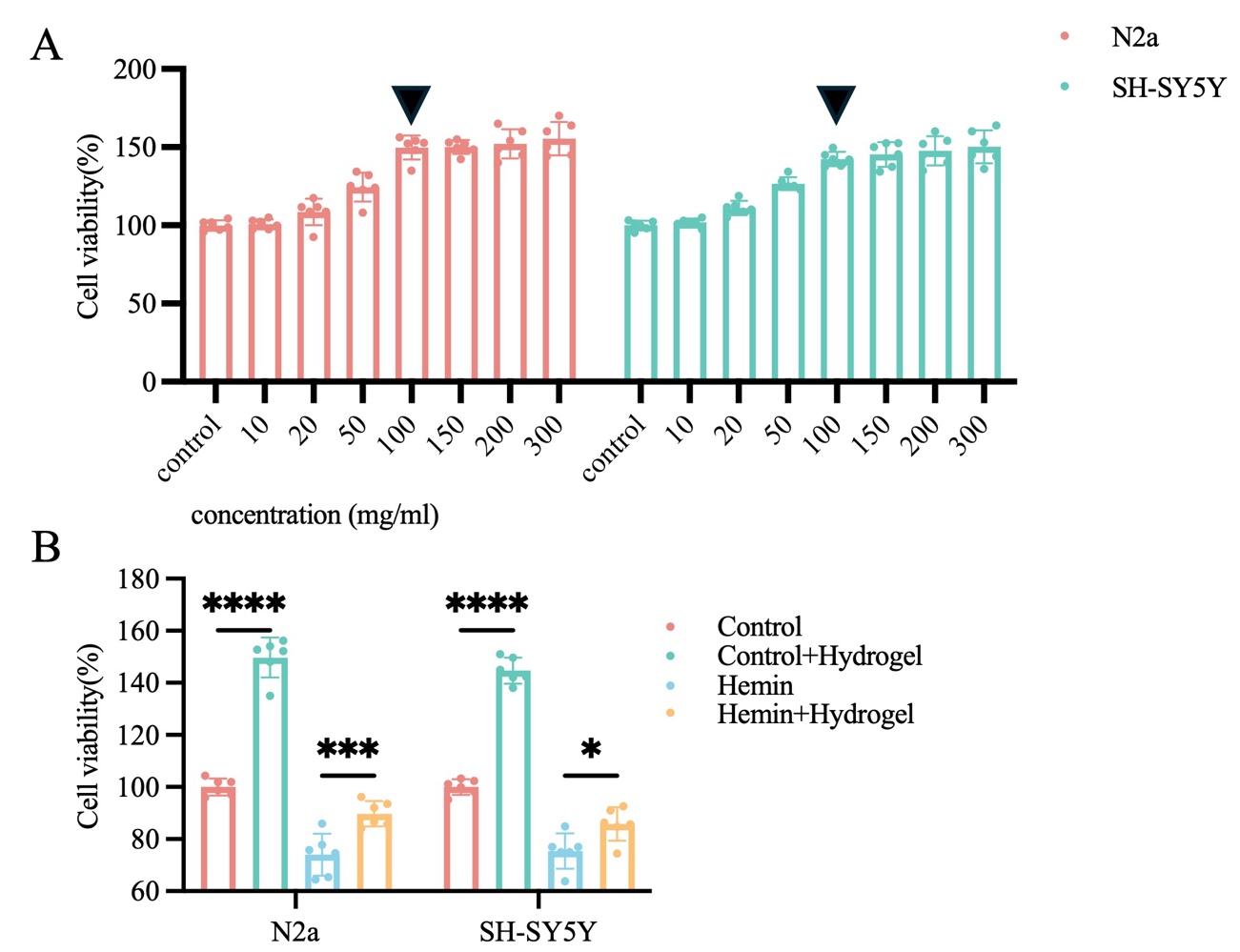


Figure S2. (A) Proliferation of N2a and SH-SY5Y cells treated with brain ECM hydrogel at different concentrations, measured by CCK-8 assay. (B) Proliferation of N2a and SH-SY5Y cells in the control, control + hydrogel, hemin, and hemin + hydrogel groups, measured by CCK-8 assay. n = 6. The black triangle represents the concentration selected for subsequent experiments. ns ＞ 0.05, **P* < 0.05, ****P* < 0.001, *****P* < 0.0001.


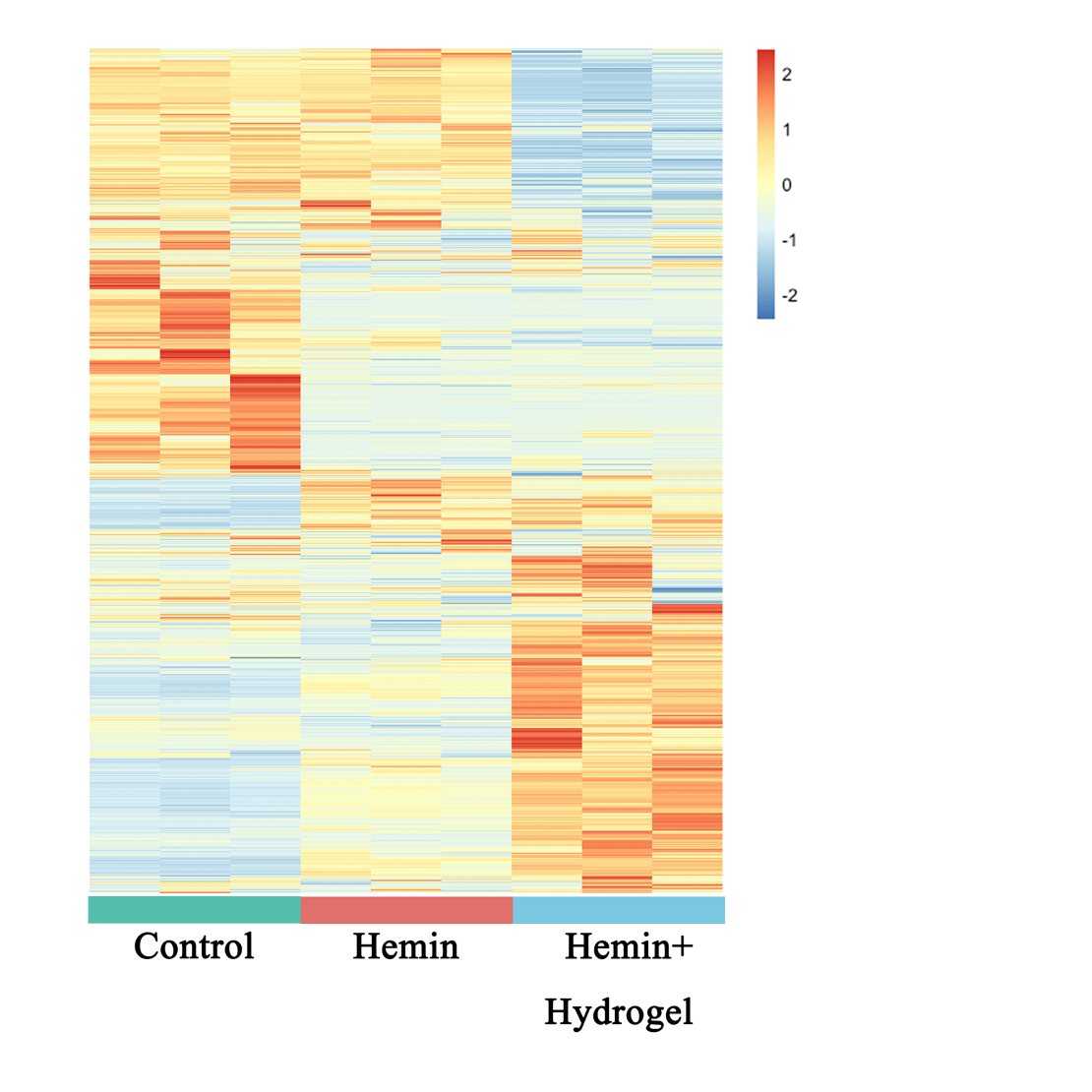


Figure S3. Heatmaps of differentially expressed mRNAs from the RNA-seq analysis of the control group, the hemin group, and the hemin + hydrogel group in N2a cells.


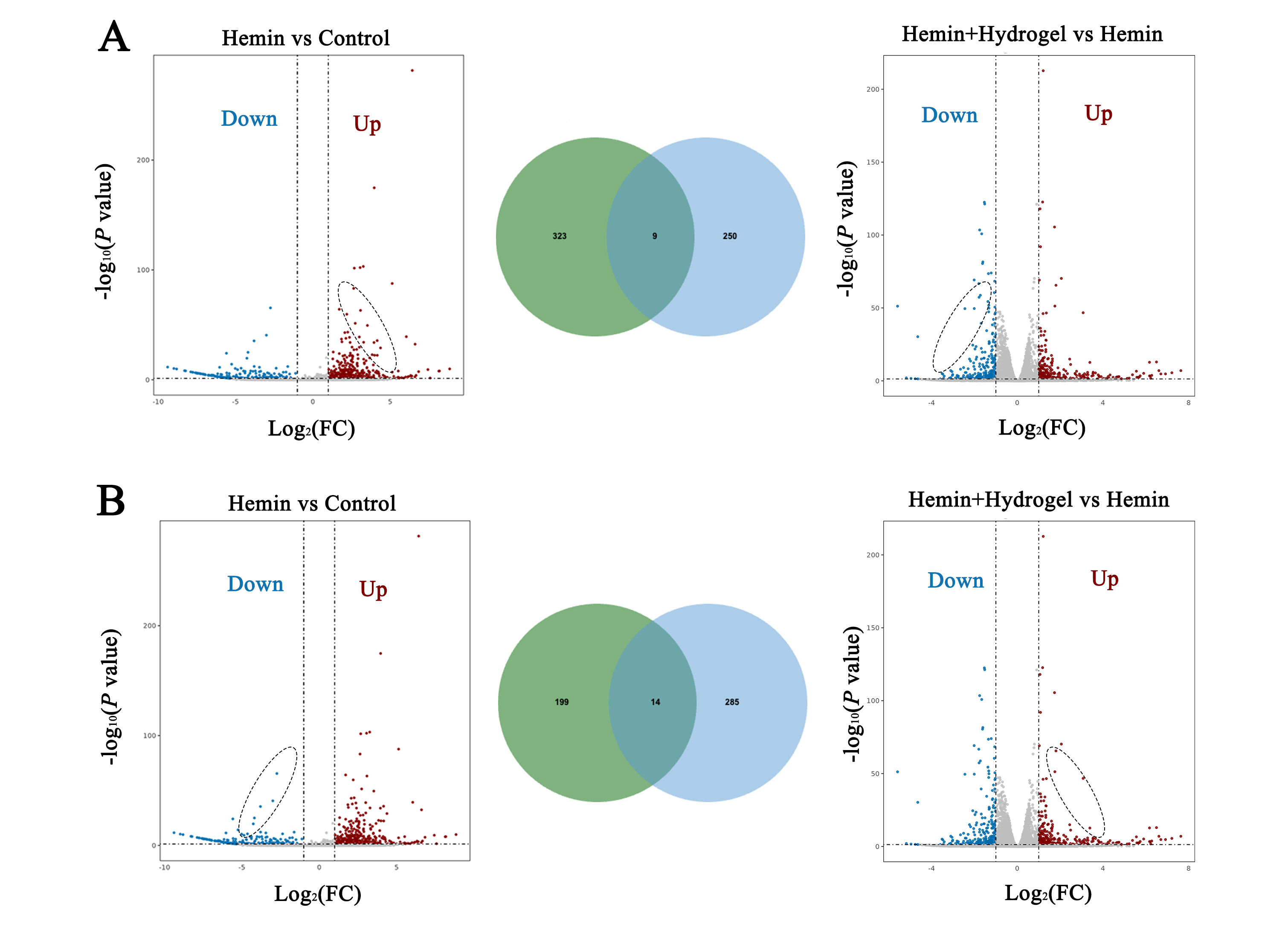


Figure S4. (A) The number of genes with higher expression (≥2-fold difference) in the hemin group compared with the control group and lower expression in the hemin + hydrogel group compared with the hemin group in N2a cells. (B) The number of genes with lower expression (≥2-fold difference) in the hemin group compared with the control group and higher expression in the hemin + hydrogel group compared with the hemin group in N2a cells.


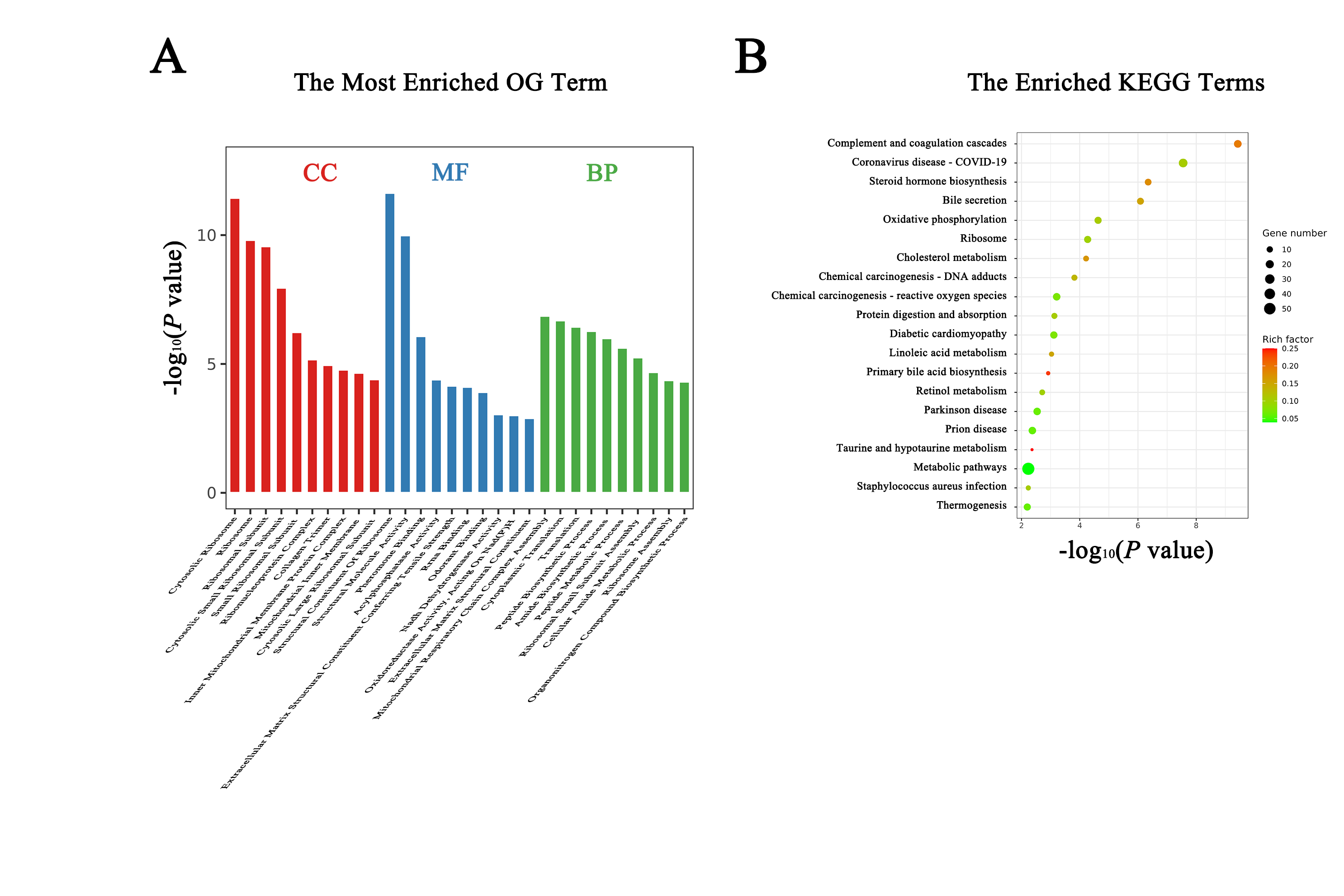


Figure S5. (A) GO enrichment analysis in the hemin + hydrogel group compared with the hemin group in N2a cells. (B) Top 20 significant KEGG pathways in the hemin + hydrogel group compared with the hemin group in N2a cells.


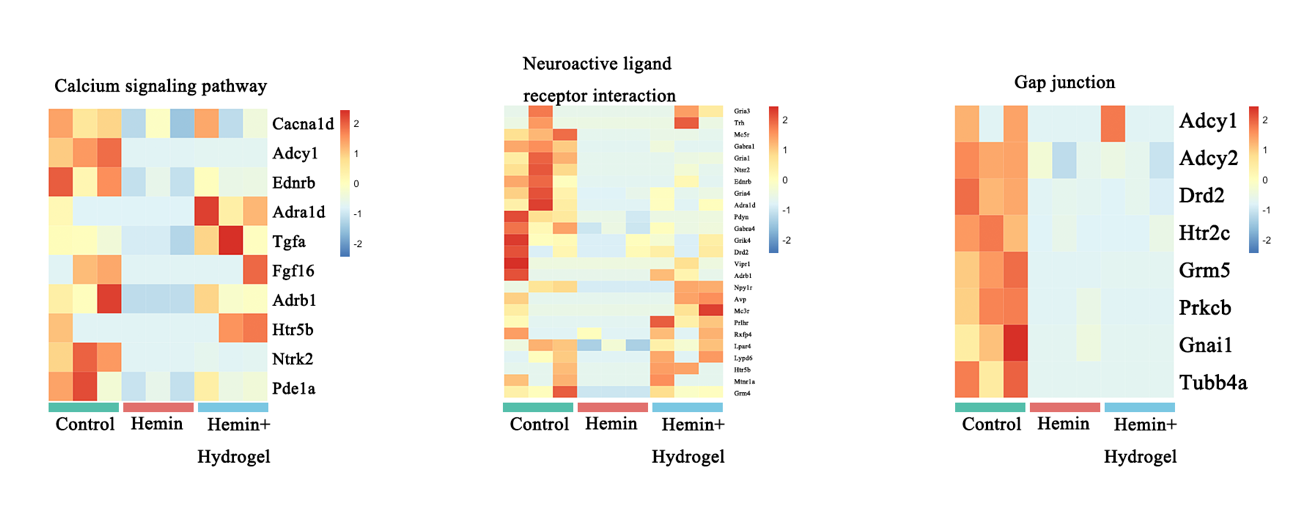


Figure S6. Heatmaps show the relative expression of genes related to calcium signaling pathway, axon guidance, neuroactive ligand-receptor interaction, gap junction, long-term potentiation, long term depression, and ribosome in the control group, the hemin group, and the hemin + hydrogel group.


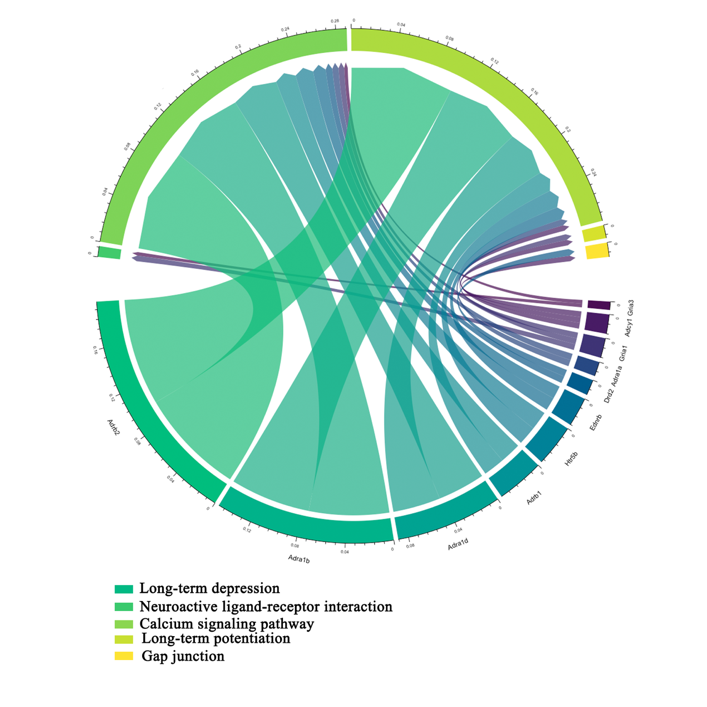


Figure S7. GOChord plot of the GO enrichment analysis in the hemin + hydrogel group compared with the hemin group.


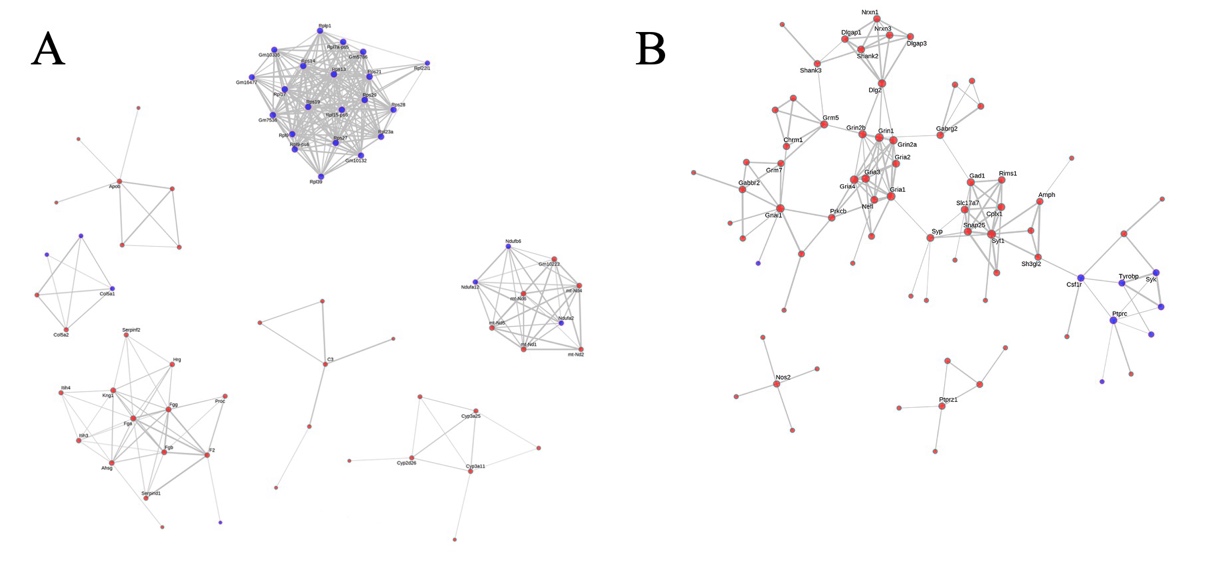


Figure S8. (A) Protein-protein interaction of DEGs between the hemin group and the hemin + hydrogel group in N2a cells. (B) Protein-protein interaction of DEGs between the hemin group and the hemin + hydrogel group in SY5Y cells.

Figure S9. (A) Immunofluorescence staining for Robo1 in the peri-cavity area. (B) semi-quantification of Robo1 fluorescence signals in the peri-cavity area (n = 6). *****P* < 0.0001.

**Supplementary tables**

Table S1. The sequences of primers used for RT-qPCR.

| primers | 5’-3’ |
| --- | --- |
| Slit2- FORWARD | GTGAATGTGAGGAAGGGTGGATGG |
| Slit2- REVERSE | GCTGTAGGAGAAGGCGTTGATGG |
| Robo1- FORWARD | AGGAGGCAGCGTGAGAGCAG |
| Robo1- REVERSE | GAGCGAGCAGGAGGAATTGGATG |

Table S2. List of primary antibodies.

| Antibody (host) | dilution ratio | Company | Cat. Ref. |
| --- | --- | --- | --- |
| SLIT2(Rabbit) | 1:1000 | proteintech | 20217-1-AP |
| ROBO1(Rabbit) | 1:1000 | proteintech | 20219-1-AP |
